# Adipsin Concentrations Are Associated with Back Pain Independently of Adiposity in Overweight or Obese Adults

**DOI:** 10.3389/fphys.2018.00093

**Published:** 2018-02-12

**Authors:** Sharmayne R. E. Brady, Aya Mousa, Negar Naderpoor, Maximilian P. J. de Courten, Flavia Cicuttini, Barbora de Courten

**Affiliations:** ^1^Department of Epidemiology and Preventive Medicine, School of Public Health and Preventive Medicine, Monash University, Melbourne, VIC, Australia; ^2^Monash Centre for Health Research and Implementation, School of Public Health and Preventive Medicine, Monash University, Clayton, VIC, Australia; ^3^Centre for Chronic Disease, Victoria University, Melbourne, VIC, Australia

**Keywords:** back pain, obesity, metabolic diseases, inflammation, adipokines

## Abstract

**Objective:** To compare cardiometabolic risk factors including cytokine and adipokine concentrations between individuals with and without back pain.

**Methods:** In 62 overweight/obese adults (BMI ≥ 25 kg/m^2^; 23F/39M), we collected data on: self-reported back pain; anthropometry [BMI, waist circumference, body composition (dual energy X-ray absorptiometry—DEXA)]; metabolic parameters [fasting glucose; insulin sensitivity (hyperinsulinaemic-euglycaemic clamps)]; cardiovascular parameters (blood pressure, lipids); serum inflammation markers [high-sensitivity C-reactive protein (hsCRP; immunoturbidimetric-assay), tumor necrosis factor-*alpha* (TNF-α), interleukin (IL)-6, and IL-10 (multiplex-assay)]; and adipokines [leptin, adipsin, resistin, and adiponectin (multiplex-assay)].

**Results:** Participants who reported having back pain in the past month (*n* = 24; 39%) had higher BMI (mean ± *SD* = 33.8 ± 6.3 vs. 30.2 ± 4.1 kg/m^2^, *p* = 0.008), fat-mass (39.9 ± 12.3 vs. 33.9 ± 9.8%, *p* = 0.04), and waist circumference (109.6 ± 16.8 vs. 101.0 ± 9.3 cm, *p* = 0.01) compared to those without back pain (*n* = 38; 61%). No differences were observed in cardiometabolic parameters, inflammatory markers, or adiponectin or resistin concentrations. Those reporting back pain had higher adipsin concentrations compared to those without back pain [median (IQR) = 744 (472–2,804) vs. 721 (515–867) ng/ml, *p* = 0.03], with a trend for higher leptin [5.5 (1.5–24.3) vs. 2.3 (1.5–6.7) ng/ml, *p* = 0.05], both of which persisted after adjustment for age and sex. Adipsin remained associated with back pain independently of adiposity (BMI, waist, fat-mass, or total %body fat; all *p* ≤ 0.03).

**Conclusions:** Greater obesity, and higher adipsin and leptin concentrations were observed in those who reported back pain in the past month compared to those without back pain, and adipsin was associated with back pain independently of adiposity. Larger studies are needed to determine if adipsin could be a novel therapeutic target for prevention and/or treatment of back pain.

## Introduction

Back pain causes greater disability worldwide than any other condition (Hoy et al., 2014), resulting in a considerable global health and financial burden (Katz, 2006). Given that there are few effective treatments for back pain (Maher et al., 2017), primary prevention strategies aiming to reduce risk factors for back pain are critical. Obesity is a potential risk factor for the development of back pain (Brady et al., 2017); however, it is unclear whether this is due to physical loading on spinal structures or systemic factors. Previous studies have shown that back pain intensity and associated disability are related to increased fat mass, but not muscle mass (Urquhart et al., 2011), raising the possibility that there may be systemic factors at play.

Other cardiometabolic risk factors may also contribute to the pathophysiology of back pain, including components of the metabolic syndrome such as hypertension, dyslipidaemia, and raised fasting blood glucose (Ranasinghe et al., 2017), but also more novel markers such as markers of inflammation (Johnson et al., 2015) and adipokines (Gomez et al., 2009). In fact, osteoarthritis has recently been labeled a metabolic disorder (Mobasheri et al., 2017), with some studies suggesting that cardiometabolic risk factors may increase risk of osteoarthritis independently of obesity (Wang et al., 2016), by dysregulating metabolic signaling, increasing oxidative stress and endothelial dysfunction, and via cholesterol accumulation in cartilage which impairs its efflux functions (Wang et al., 2016).

It is also proposed that pain in osteoarthritis occurs via inflammatory mechanisms (Mobasheri et al., 2017) with a potential role for adipokines (Martel-Pelletier et al., 2016). Adipose tissue secretes many different substances, including cytokines, such as interleukins (IL), tumor necrosis factor-*alpha* (TNF-α), and adipokines such as adiponectin, leptin, and adipsin (Pottie et al., 2006; Robinson et al., 2009). These inflammatory markers and adipokines may represent future therapeutic targets (Johnson et al., 2015). Indeed, leptin has been proposed as a possible causative link between obesity and osteoarthritis (Vuolteenaho et al., 2014; Martel-Pelletier et al., 2016), while TNF-α and ILs-6 and−8 have been implicated in structural joint abnormalities (Dumond et al., 2003), nociceptive pathways (Lübbeke et al., 2013), and in the development and progression of chronic pain (Dina et al., 2008; Wang et al., 2008). However, previous studies are limited by small sample sizes and/or inclusion of patients with existing osteoarthritic diseases or who were undergoing surgery (Wang et al., 2008; Kraychete et al., 2010; Queiroz et al., 2015). To our knowledge, no studies have explored novel adipokines such as adipsin or resistin in relation to back pain, and inconsistent findings have been reported for relationships between back pain and other adipokines such as leptin and adiponectin (Shiri et al., 2008; Lippi et al., 2017). Consequently, important knowledge gaps remain regarding whether cardiometabolic risk factors including inflammatory markers and adipokines contribute to back pain, particularly in high-risk groups such as overweight or obese adults.

We aimed to address this knowledge gap by examining relationships between back pain and cardiometabolic risk factors including cytokines and adipokines in a metabolically well-characterized cohort, using gold-standard measurements of adiposity and insulin sensitivity. Specifically, we aimed to determine whether overweight or obese individuals with back pain within the past month had poorer cardiometabolic profiles and/or unfavorable concentrations of cytokines and/or adipokines compared to individuals without back pain.

## Materials and methods

### Design and participants

This cross-sectional study utilizes baseline data from a previous randomized controlled trial (RCT) which examined the effects of vitamin D supplementation on insulin sensitivity in overweight or obese adults, with methods reported previously (de Courten et al., 2015). Briefly, volunteers were recruited from the community using online and poster advertising in Melbourne, Australia, and were included if they were adults aged 18–60 years with a BMI ≥ 25 kg/m^2^ and a stable weight (<5 kg change in preceding year). Participants were not selected based on back pain status, and were excluded if they were smokers, had high alcohol consumption, or used any medications (including pain medications), vitamins, or supplements. Participants with hypercalcaemia, allergies, diabetes, or any medical condition, central nervous system or psychiatric disorders, active cancer within the preceding 5 years, or the presence of acute inflammation were excluded. Pregnant, lactating, or peri/post-menopausal women were excluded. The study was carried out in accordance with the recommendations of the Declaration of Helsinki with written informed consent from all participants. The protocol was approved by the Monash University Human Research Ethics Committee (CF13/3874-2013001988).

### Self-reported measures including back pain

All measurements were performed at three visits attended over 1 week. At the first visit, participants underwent a medical history and examination which included the question “Have you had back pain in the past month?” Participant responses as “yes” or “no,” were recorded and used to determine back pain status in the present study. Data on physical activity was collected using the validated International Physical Activity Questionnaire (IPAQ), which calculates multiples of the resting metabolic rate (METs) to characterize the level of exercise intensity, wherein a single MET represents the energy utilized by the body at rest.

### Anthropometric and cardiometabolic assessments

Body mass index (BMI) was also calculated at the first visit using weight (kg)/height (m)^2^, and waist circumference was measured as an additional measure of central obesity. At the second visit, dual energy X-ray absorptiometry (DEXA) scans, the gold-standard measure of adiposity, were performed to determine body fat composition as total percentage body fat. Total body fat was then used to calculate fat mass [weight (kg) × total % body fat (decimal) = fat mass (kg)] as well as fat-free mass [weight (kg) – fat mass (kg) = fat-free mass (kg)]. Resting systolic and diastolic blood pressure (SBP, DBP) were measured after a 20-min rest, using an automated oscillometric measurement system (M6 Automatic BP monitor, Omron, Japan), and the average of three measurements was recorded. Insulin sensitivity was measured at the third visit using the gold-standard hyperinsulinaemic-euglycaemic clamp as previously described (de Courten et al., 2015).

### Biochemical analyses

Fasting venous blood samples were collected and analyzed under blinded conditions by an accredited and quality-assured laboratory (Monash Health Pathology, Melbourne). Serum triglycerides, and total, low-, and high-density lipoprotein (LDL/HDL) cholesterol were measured using commercial enzymatic assays with inter- and intra-assay CVs <8% (LX20PRO Analyser, SYNCHRON Multi-calibrators; Beckman Coulter Inc., Sydney, Australia). Fasting blood glucose was measured after a 12 h overnight fast using the glucose oxidase method (YSI-2300 STAT; YSI Inc., OH, USA).

High sensitivity C-reactive protein (hsCRP) concentrations were measured using highly sensitive near-infrared particle immunoassay on a Synchron LX System Analyser according to manufacturer's instructions (Beckman Coulter Inc., Sydney, Australia), with inter- and intra-assay CVs of <3 and <5%, respectively. Inflammatory markers and adipokines were measured using bead-based multi-analyte assays (LEGENDplex™ Human Inflammation and Human Metabolic Panels; Biolegend, San Diego, CA). These panels simultaneously quantified pro- and anti- inflammatory markers including TNF-α, IL-6, and IL-10, and adipokines including leptin, adiponectin, adipsin, and resistin. Data were analyzed using the LEGENDplex™ Data Analysis Software (BioLegend, San Diego, CA) with standard curves generated from 0 to 50,000 pg/ml for inflammatory markers and 0 to 200 ng/ml for adipokines, and samples adjusted for dilution factors. Inter- and intra-assay CVs for all analytes were <8 and <9%, respectively.

### Statistical analyses

Data for this study was derived from a previous RCT where the sample size calculation has been reported elsewhere (de Courten et al., 2015), and was based on insulin sensitivity as the primary outcome. Results are presented as mean ± standard deviation (*SD*) or median (interquartile range) unless otherwise specified. Normality was assessed by visual inspection of histograms and Shapiro-Wilk tests and non-normally distributed variables were transformed as appropriate (logarithmic or square root transformations) to approximate normality prior to analyses. Independent samples *t*-tests and chi-squared tests were used to compare continuous and categorical variables, respectively, between those with and without back pain. Multivariable logistic regression was used to examine differences between those with and without back pain after adjusting for clinically relevant variables including age, sex, and measures of adiposity. Interaction terms for gender were added to the multivariable models to examine whether differences between groups (back pain vs. no back pain) varied by gender. Findings were considered statistically significant at a two-tailed level of *P* < 0.05. All analyses were performed using Stata V.13 (StataCorp, TX, USA).

## Results

### Sample characteristics

Sixty-two participants (39 males/23 females) were included in the analysis, with a mean age of 31.3 ± 8.5 years and a mean BMI and total % body fat of 31.6 ± 5.3 kg/m^2^ and 40.2 ± 8.7%, respectively. There were no differences between genders in age (*p* = 0.6) or BMI (*p* = 0.08), however, females had a higher total % body fat (49.0 ± 5.5 vs. 35.3 ± 5.8%, respectively, *p* < 0.001) and higher fat mass (40.5 ± 8.0 vs. 33.7 ± 11.9 kg, respectively, *p* = 0.02) than males, while males had a higher fat-free mass (60.0 ± 10.5 vs. 41.7 ± 5.7 kg, respectively, *p* < 0.001). Males had an overall poorer cardiometabolic profile, with higher fasting glucose concentrations (4.7 ± 0.5 vs. 4.4 ± 0.5 mmol/l, *p* = 0.03), SBP (125.1 ± 12.1 vs. 115.4 ± 2.2 mmHg, *p* = 0.002), and triglycerides (1.8 ± 1.0 vs. 1.2 ± 0.4 mmol/l, *p* = 0.004) compared to females, respectively. Leptin concentrations were higher in females compared to males (33.4 ± 41.3 vs. 4.7 ± 7.5 ng/ml, respectively, *p* < 0.001), however there were no differences in the other adipokines or serum inflammatory markers (all *p* > 0.1).

Demographic, anthropometric, and metabolic characteristics of participants with and without back pain in the past month are presented in Table 1. Twenty-four (39%) of participants reported having back pain in the past month while the remaining 38 (61%) participants reported no back pain. Age and gender of participants as well as physical activity (IPAQ-METs) were not significantly different between those with and without back pain (both *p* > 0.1; Table 1).

**Table 1:** Univariable and multivariable analyses for differences in demographic, anthropometric, and cardiometabolic parameters between those with and without back pain in the past month.

**Characteristics**	**Back pain in the last month (*n* = 24)**	**No back pain in the past month (*n* = 38)**	***p*_1_**	**or (95% CI)**	***p*_2_**
Age (years)	30.3 ± 8.2	32.0 ± 8.8	0.4	–	–
Females, *n* (%)	10 (41.7)	13 (34.2)	0.6	–	–
IPAQ-METs score	1,665 (735–3,474)	2,375 (1,017–4,851)	0.7	1.0 (0.9–1.0)	0.6
**ANTHROPOMETRIC PARAMETERS**					
Body mass index (kg/m^2^)	32.6 (29.1–37.0)	29.7 (27.6–31.5)	**0.01**	1.2 (1.0–1.3)	**0.02**
Waist circumference (cm)	109.6 ± 16.9	101.0 ± 9.3	**0.01**	1.1 (1.0–1.1)	**0.02**
Total body fat (%)	41.8 ± 9.3	39.3 ± 8.3	0.3	1.1 (0.9–1.2)	0.2
Fat mass (kg)	39.9 ± 12.3	33.9 ± 9.8	**0.04**	1.1 (1.0–1.1)	0.05
Fat-free mass (kg)	55.5 ± 15.1	52.1 ± 10.9	0.3	1.1 (1.0–1.1)	0.08
**CARDIOMETABOLIC PARAMETERS**					
Fasting glucose (mmol/l)	4.7 ± 0.6	4.5 ± 0.5	0.2	2.4 (0.8–7.3)	0.1
Insulin sensitivity (mg/kg/min)	6.3 ± 3.1	6.6 ± 2.7	0.7	1.0 (0.8–1.2)	0.9
Systolic blood pressure (mmHg)	121.4 ± 14.5	121.6 ± 11.2	0.9	1.0 (0.9–1.1)	0.6
Diastolic blood pressure (mmHg)	80.9 ± 9.4	80.2 ± 8.5	0.8	1.0 (0.9–1.1)	0.6
Total cholesterol (mmol/l)	4.9 ± 0.9	5.0 ± 0.9	0.7	1.0 (0.5–1.7)	0.9
Triglycerides (mmol/l)	1.5 ± 0.6	1.6 ± 1.0	0.4	0.6 (0.1–8.1)	0.7
HDL cholesterol (mmol/l)	1.2 ± 0.3	1.2 ± 0.2	0.5	2.4 (0.3–19.6)	0.4
LDL cholesterol (mmol/l)	3.0 ± 0.9	3.1 ± 0.7	0.6	0.9 (0.4–1.9)	0.8
**INFLAMMATORY MARKERS AND ADIPOKINES**					
hsCRP (mg/l)	3.0 (1.0–6.0)	1.4 (0.8–3.5)	0.3	1.9 (0.6–5.9)	0.2
TNF-α (pg/ml)	30.3 (16.3–60.4)	34.8 (21.2–72.9)	0.7	0.7 (0.2–2.2)	0.6
IL-6 (pg/ml)	27.2 (9.5–43.4)	21.2 (13.5–44.4)	0.8	0.8 (0.3–2.2)	0.6
IL-10 (pg/ml)	9.3 (6.4–13.6)	8.4 (6.8–17.0)	0.7	0.5 (0.1–3.9)	0.5
Adiponectin (ng/ml)	7,713 (2,885–14,516)	3,849 (2,348–9,915)	0.3	1.7 (0.7–4.5)	0.3
Adipsin (ng/ml)	744 (472–2,804)	721 (515–867)	**0.04**	1.1 (1.0–1.1)	**0.03**
Leptin (ng/ml)	5.5 (1.5–24.3)	2.3 (1.5–6.7)	0.05	3.2 (0.9–10.2)	0.05
Resistin (ng/ml)	0.4 (0.3–1.4)	0.5 (0.3–0.9)	0.9	1.0 (0.9–1.0)	0.9

*Data presented as mean ± SD or median (IQR) for non-normally distributed variables, unless otherwise specified. Non-normally distributed variables underwent logarithmic or square root transformation to approximate normality prior to analyses. Bold figures indicate statistical significance. **P**_1_ = chi-squared tests or independent Student's t-tests for differences between groups in categorical and continuous variables, respectively; **P**_2_ = logistic regression adjusted for age and sex with back pain as dependent variable. IPAQ-METs, international physical activity questionnaire-multiples of the resting metabolic rate; HDL/LDL, high/low-density lipoprotein; hsCRP, high sensitivity C-reactive protein; TNF-α, tumor necrosis factor-alpha; IL, interleukin*.

### Back pain and anthropometric and cardiometabolic parameters

Those who reported back pain in the past month had a significantly higher BMI (*p* = 0.01), waist circumference (*p* = 0.01), and fat mass (*p* = 0.04) compared to those without back pain (Table 1). There were no differences between groups in metabolic parameters including fasting glucose concentrations or insulin sensitivity (both *p* > 0.1; Table 1). Similarly, SBP and DBP as well as lipid profiles did not differ between those with and without back pain in the past month (all *p* > 0.1; Table 1).

After adjustment for age and sex, higher BMI and waist circumference remained significantly associated with back pain (*p* = 0.02 for both), and trends were observed for higher fat mass (*p* = 0.05) and fat-free mass (*p* = 0.08) (Table 1). Associations between cardiometabolic parameters and back pain remained non-significant after adjusting for age and sex (all *p* ≥ 0.1, Table 1). Additional adjustment for fat mass, BMI, waist circumference, or total % body fat did not alter the results (all *p* ≥ 0.1; data not shown). There were no gender differences in the relationships between any of the anthropometric or cardiometabolic parameters and back pain (all *p* > 0.1 for gender interactions; data not shown).

### Back pain and serum inflammatory markers and adipokines

There were no differences between those with or without back pain in the past month in cytokine concentrations including hsCRP (*p* = 0.2), TNF-α (*p* = 0.6), IL-6 (*p* = 0.6), or IL-10 (*p* = 0.5) (Table 1). Results remained non-significant after adjusting for age and sex (all *p* > 0.1; Table 1), as well as after further adjustment for any of the adiposity measures (all *p* > 0.1; data not shown). There was no significant interaction by gender in the relationships between back pain and any of these markers (all *p* > 0.1 for interactions; data not shown).

**Figure 1:**
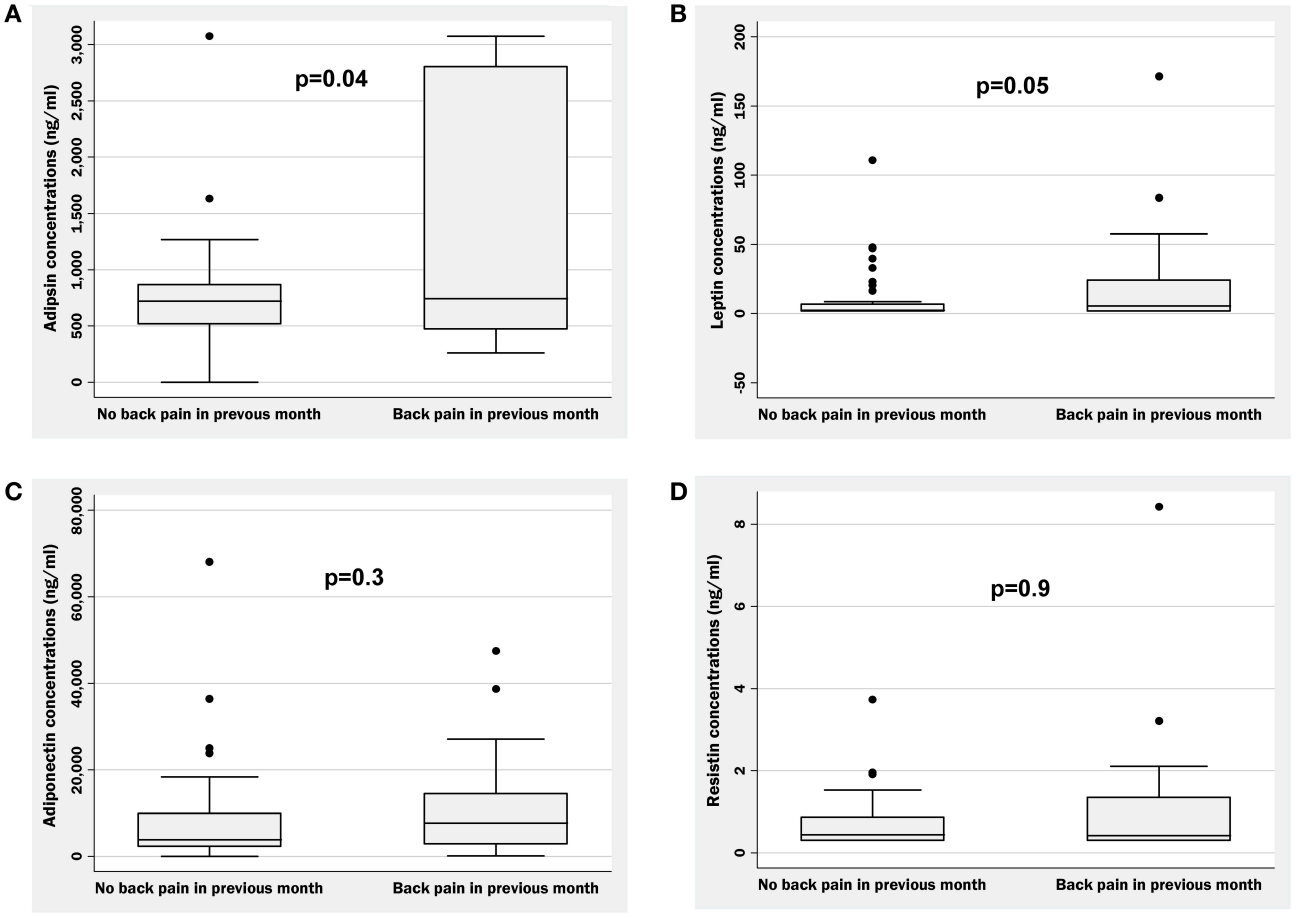
Boxplots illustrating raw data for concentrations of adipsin **(A)**, leptin **(B)**, adiponectin **(C)**, and resistin **(D)** in those with and without self-reported back pain in the previous month. *p* = differences between groups using simple logistic regression after logarithmic or square root transformation to approximate normality.

Those who reported back pain in the past month had significantly higher adipsin concentrations compared to those reporting no back pain [median (IQR) = 744 (472–2,804) vs. 721 (515–867) ng/ml, respectively, *p* = 0.04; Table 1, Figure 1A]. Associations between adipsin and back pain remained significant after adjusting for age and sex (*p* = 0.03; Table 1) as well as after additional adjustment for any of the adiposity measures including BMI, waist circumference, fat mass, or total % body fat (all *p* ≤ 0.03; Table 2). In models which included age, sex, and BMI or waist circumference, both BMI or waist circumference as well as adipsin levels were associated with back pain (all *p* < 0.05; data not shown).

**Table 2:** Multiple logistic regression models exploring relationships between adipokines and back pain after adjustment for age, sex, and adiposity measures.

**Dependent variable: Back pain in the past month**												
**Independent variables**	**Model 1 adjusted for age, sex, and BMI**			**Model 2 adjusted for age, sex, and waist circumference**			**Model 3 adjusted for age, sex, and fat mass**			**Model 4 adjusted for age, sex, and total % body fat**		
	**OR (95% CI)**	**SE**	***P***	**OR (95% CI)**	**SE**	***P***	**OR (95% CI)**	**SE**	***P***	**OR (95% CI)**	**SE**	***P***
Leptin (ng/ml)	2.0 (0.6–7.1)	1.3	0.3	1.8 (0.5–6.7)	1.2	0.4	2.04 (0.6–7.4)	1.3	0.3	2.6 (0.7–9.1)	1.7	0.1
Adipsin (ng/ml)	1.1 (1.01–1.11)	0.03	**0.02**	1.1 (1.01–1.11)	0.03	**0.03**	1.1 (1.01–1.11)	0.03	**0.03**	1.1 (1.01–1.11)	0.03	**0.03**
Adiponectin (ng/ml)	1.5 (0.6–4.2)	0.8	0.4	1.6 (0.6–4.6)	0.9	0.4	1.5 (0.5–4.0)	0.8	0.5	1.6 (0.6–4.3)	0.8	0.4
Resistin (ng/ml)	1.0 (0.97–1.03)	0.02	0.9	1.0 (0.97–1.03)	0.02	0.9	1.0 (0.97–1.03)	0.02	0.9	1.0 (0.97–1.03)	0.02	0.9

*Dependent variable: back pain in the past month (binary). Data reported as odds ratios with 95% confidence intervals [OR (95% CI)] with corresponding standard errors (SE) and p-values. Non-normally distributed variables underwent logarithmic or square root transformation to approximate normality prior to analyses. BMI, body mass index. Bold figures indicate statistical significance*.

A trend for higher leptin concentrations was observed in participants who reported back pain in the past month compared to those reporting no back pain [5.5 (1.5–24.3) vs. 2.3 (1.5–6.7), respectively, *p* = 0.05; Table 1, Figure 1B]. This trend persisted after adjustment for age and sex (*p* = 0.05; Table 1), but was attenuated after further adjustment for BMI, waist circumference, fat mass, or total % body fat (all *p* ≥ 0.1; Table 2). In models which included age, sex, measures of adiposity, and leptin concentrations, neither leptin concentrations nor adiposity measures were associated with back pain (all *p* > 0.05; data not shown).

Adiponectin and resistin concentrations did not differ between groups in unadjusted analysis (*p* = 0.3 and *p* = 0.9, respectively; Figures 1C,D) as well as after adjustment for age and sex (*p* = 0.3 and *p* = 0.9, respectively; Table 1). Additional adjustment for fat mass, BMI, waist circumference or total % body fat did not alter the results (all *p* > 0.1; Table 2). There were no gender differences in the relationships between any of the adipokines and back pain (all *p* > 0.1 for gender interactions; data not shown).

## Discussion

We report an association between serum adipsin concentrations and self-reported back pain in the past month which was independent of adiposity measures including BMI, waist circumference, fat mass, or total % body fat. To our knowledge, this is the first study to report an association between adipsin and back pain in a metabolically well-characterized cohort of overweight or obese, otherwise healthy adults. We also report a trend for a relationship between leptin and back pain which was attenuated after adjustment for adiposity. We confirm previous studies that obesity is associated with back pain; however no associations were observed between back pain and cardiometabolic risk factors. Our findings suggest that adipsin may be related to back pain independently of adiposity and further studies are needed to determine if adipsin could be a novel therapeutic target for prevention and/or treatment of back pain.

We report that individuals with back pain in the past month had higher BMI, waist circumference, and fat mass compared to those without back pain. This is consistent with previous studies that have also shown greater levels of adiposity in those with back pain (Urquhart et al., 2011; Chou et al., 2016). There were no significant differences in cardiometabolic parameters between those with and without back pain in the past month. Few studies have examined the relationship between cardiometabolic risk factors or the metabolic syndrome and back pain (Ha, 2011; Duruöz et al., 2012; Suri et al., 2012; Teraguchi et al., 2016). In a Japanese study of 928 adults, those with one or more components of the metabolic syndrome had higher odds of thoracic, but not lumbar spine, disc degeneration determined by MRI, compared to those without any metabolic syndrome components (Teraguchi et al., 2016). Conversely, in 435 participants from the Framingham Heart Study, diabetes, hypercholesterolemia, and hypertension were not associated with disc height loss on CT (Suri et al., 2012). However, both studies did not collect data on back pain symptoms. Another study of 60 patients with lower back pain for ≥ 2 months found that 25% had the metabolic syndrome (Duruöz et al., 2012). Similarly, in 1,085 Korean patients with chronic low back pain, the prevalence of metabolic syndrome was relatively high at 36.2% (Ha, 2011). Neither of these studies included participants without back pain, hence no comparisons were made regarding the prevalence of cardiometabolic risk factors between those with and without back pain. Here, we show that in a well-characterized cohort of young, healthy adults, there were no differences in cardiometabolic risk factors between those with and without back pain in the past month.

We found no differences in inflammatory markers including hsCRP, TNF-α, or IL-6 or −10 between those with or without back pain in the past month. A population-based study using National Health and Nutrition Examination Survey data from 15,322 people demonstrated that elevated CRP (>3.0 mg/dL) was associated with greater odds of back pain, the odds further increasing in those who were obese (Briggs et al., 2013). Similarly, a cross-sectional study of 2,575 young adults found that higher CRP was associated with an increased prevalence of back pain in women (Shiri et al., 2008). Previous studies have also reported elevated pro-inflammatory cytokines such as ILs-6, −7, and −8 in tissues of patients undergoing surgery for back pain (Altun, 2016; Zhang et al., 2016); associations between TNF-α, IL-6, and IL-8 with back pain (Wang et al., 2008; Brinkley et al., 2009; Kraychete et al., 2010; Pedersen et al., 2015; Queiroz et al., 2017); and a possible key role for TNF-α and IL-1β in the pathology of intervertebral disc degeneration (Li et al., 2014). Whilst our study showed no differences in inflammatory marker concentrations between those with or without back pain, this was likely due to our small sample size and possible lack of power to detect a significant difference. Notably, our study was in healthy young adults, while some of the previous studies were in elderly women (age ≥ 65 years) (de Queiroz et al., 2016; Queiroz et al., 2017), who due to their advanced age, may have higher levels of cytokines and inflammation (Franceschi et al., 2007). Other studies were limited to participants with existing chronic diseases or significant disability (Wang et al., 2008; Brinkley et al., 2009), those undergoing surgery for back pain (Altun, 2016; Zhang et al., 2016), or who were recruited from large tertiary-care hospitals (Pedersen et al., 2015). These populations may have elevated levels of inflammatory markers which may, in part, explain the discrepancy with our findings since participants in our study were healthy individuals without existing comorbidities or underlying inflammation.

We report the novel finding that back pain in the past month was associated with higher adipsin concentrations, with a trend also observed for a relationship with higher leptin concentrations. Adipokines such as leptin and adipsin are reported to be involved in appetite and weight maintenance, but also have inflammatory and nociceptive properties (Fantuzzi, 2005; Lim et al., 2009). Leptin is thought to have a role in neuropathic pain (Lim et al., 2009) and pain in knee, hip, and hand osteoarthritis (Clockaerts et al., 2010; Massengale et al., 2012; Lübbeke et al., 2013). Increased leptin is directly associated with obesity and is suspected to be involved in reorganizing the cytoskeleton of nucleus pulposus cells, thereby altering disc organization and structure (Samartzis et al., 2013). Indeed, *in vitro* studies have reported a role for leptin in disc degeneration (Zhao et al., 2008; Samartzis et al., 2013; Segar et al., 2016), and leptin is proposed as a causative link between obesity and osteoarthritis (Hui et al., 2012; Vuolteenaho et al., 2014). However, few epidemiological studies have explored the association between leptin and back pain, with inconsistent findings. In a study of 104 patients with back pain and 52 healthy controls, those with back pain had lower leptin levels compared to controls (Lippi et al., 2017). Conversely, a larger cross-sectional study of 2,575 young Finnish adults found that the prevalence of back pain increased with increasing serum leptin in women (Shiri et al., 2008). Here, we report a trend for increased leptin levels in those with back pain, which is attenuated after adjustment for adiposity. Our findings suggest that associations between leptin and back pain may be a result of underlying adiposity, particularly fat mass, which has seldom been measured in previous studies (Shiri et al., 2008). Further large-scale studies are needed to confirm the direction and magnitude of the relationship between leptin and back pain, and to determine whether this relationship may be mediated by adiposity.

In contrast, our findings showed that adipsin was associated with back pain in the past month independently of any measure of adiposity. Although thought to have pro-inflammatory properties, adipsin has been poorly studied in relation to bone health and osteoarthritis. One study which measured adipsin and leptin in 138 patients found that both adipsin and leptin levels predicted greater knee cartilage volume loss over time, and were associated with higher incidence of total knee replacement (Martel-Pelletier et al., 2016). Authors hypothesized that joint inflammation in knee osteoarthritis may induce an increase in leptin concentrations and, secondarily, catabolic factors such as adipsin, leading to cartilage degradation and loss (Martel-Pelletier et al., 2016). Similarly, in genomic studies, higher adipsin levels were associated with moderate and severe osteoarthritis (Fernández-Puente et al., 2011). Adipsin is known to act as a serine protease (regulator of bone remodeling) and to be a component of the alternative complement pathway (which activates innate immune responses) (Martel-Pelletier et al., 2016); however, no study has yet reported its biological or molecular effects in articular tissues. Moreover, to our knowledge, no studies have examined the relationship between adipsin and back pain. Here, we show for the first time that higher adipsin levels were related to back pain in overweight or obese, otherwise healthy adults, independently of adiposity, which is consistent with previous studies of higher adipsin in knee osteoarthritis (Martel-Pelletier et al., 2016). Collectively, these findings suggest that adipsin may be involved in the pathophysiology of osteoarthritis or back pain through a systemic inflammatory pathway unrelated to obesity. However, further studies are needed to confirm the role of adipsin in back pain and to determine its underlying mechanisms.

This study has some limitations. The cross-sectional nature of the study means we cannot establish causality or determine whether changes in anthropometric or cardiometabolic parameters may affect back pain longitudinally. Because the data used was from a previous RCT, back pain was not the primary outcome and there was no formal power calculation. Hence, our sample size was likely underpowered to detect differences in cardiometabolic parameters or inflammatory markers such as hsCRP between those with or without back pain. The study was not designed to assess back pain as a primary outcome and we were only able to determine back pain using a single question. This limits the precision of the study in determining the severity or degree of disability caused by back pain, or in defining subgroups of participants with severe or chronic back pain. Use of a validated questionnaire such as the Roland-Morris Disability Questionnaire or the Waddell Disability Index may therefore have yielded different results. The sample comprised overweight or obese, otherwise healthy adults, hence we were unable to compare our findings between overweight/obese and lean individuals and our results may not be generalizable to other populations including lean adults or those with existing comorbidities or osteoarthritic conditions.

Nevertheless, this study is to our knowledge the first to report a relationship between back pain and novel adipokines such as adipsin. We were able to show that adipsin was associated with back pain independently of adiposity, which has not been previously reported. We included a well-characterized cohort of overweight or obese, otherwise healthy adults, where there was no confounding by diseases status, comorbidities, or medication use. Gold-standard measures of cardiometabolic risk were employed, including DEXA for body composition, and hyperinsulinaemic-euglycaemic clamp for insulin sensitivity.

To conclude, despite higher rates of obesity in those with back pain, we found similar cardiometabolic risk profiles between those with and without back pain in the past month. However, adipsin and leptin concentrations were higher in those with back pain, and adipsin was associated with back pain independently of adiposity. Our findings provide new insights into the metabolic and inflammatory profiles of those with back pain, and highlight the need for larger studies to determine if adipsin could be a novel therapeutic target for prevention and/or treatment of back pain.

## Author contributions

SB: Performed data analysis and interpretation and wrote the first draft of the manuscript. AM: Performed data collection, analysis, and interpretation and wrote the first draft of the manuscript. NN: Performed data collection and contributed to data interpretation and writing and editing the manuscript. MdC: Contributed to study design and obtaining funding, as well as data interpretation and writing and editing the manuscript. FC: Contributed to data interpretation and writing and editing the manuscript. BdC: Is the chief investigator of the study and planned and designed the study, obtained funding, oversaw data collection and analysis, and supervised data interpretation and writing and editing of the manuscript. BdC: Is the guarantor of this work and takes responsibility for data integrity and accuracy. All authors meet the ICMJE criteria for authorship and have provided important intellectual input and approved the final version of the manuscript.

### Conflict of interest statement

The authors declare that the research was conducted in the absence of any commercial or financial relationships that could be construed as a potential conflict of interest.

## References

[B1] Altun I. (2016). Cytokine profile in degenerated painful intervertebral disc: variability with respect to duration of symptoms and type of disease. Spine J. 16, 857–861. 10.1016/j.spinee.2016.03.019 26975459

[B2] Brady S. R. E. Monira Hussain S. Brown W. J. Heritier S. Wang Y. Teede H.. (2017). Predictors of back pain in middle-aged women: data from the Australian longitudinal study of women's health. Arthritis Care Res. 69, 709–716. 10.1002/acr.22982 27390116

[B3] Briggs M. S. Givens D. L. Schmitt L. C. Taylor C. A. (2013). Relations of C-reactive protein and obesity to the prevalence and the odds of reporting low back pain. Arch. Phys. Med. Rehabil. 94, 745–752. 10.1016/j.apmr.2012.11.026 23187041

[B4] Brinkley T. E. Leng X. Miller M. E. Kitzman D. W. Pahor M. Berry M. J. (2009). Chronic inflammation is associated with low physical function in older adults across multiple comorbidities. J. Gerontol. A Biol. Sci. Med. Sci. 64A, 455–461. 10.1093/gerona/gln038 PMC2657165 19196644

[B5] Chou L. Brady S. R. Urquhart D. M. Teichtahl A. J. Cicuttini F. M. Pasco J. A.. (2016). The association between obesity and low back pain and disability is affected by mood disorders: a population-based, cross-sectional study of men. Medicine 95:e3367. 10.1097/MD.0000000000003367 27082599 PMC4839843

[B6] Clockaerts S. Bastiaansen-Jenniskens Y. M. Runhaar J. Van Osch G. J. Van Offel J. F. Verhaar J. A.. (2010). The infrapatellar fat pad should be considered as an active osteoarthritic joint tissue: a narrative review. Osteoarthr. Cartil. 18, 876–882. 10.1016/j.joca.2010.03.014 20417297

[B7] de Courten B. Mousa A. Naderpoor N. Teede H. de Courten M. P. Scragg R. (2015). Vitamin D supplementation for the prevention of type 2 diabetes in overweight adults: study protocol for a randomized controlled trial. Trials 16:335. 10.1186/s13063-015-0851-6 26246241 PMC4527336

[B8] de Queiroz B. Z. Pereira D. S. Lopes R. A. Felício D. C. Silva J. P. Rosa N. M.. (2016). Association between the plasma levels of mediators of inflammation with pain and disability in the elderly with acute low back pain: data from the Back Complaints in the Elders (BACE)-Brazil study. Spine 41, 197–203. 10.1097/BRS.0000000000001214 26571172

[B9] Dina O. A. Green P. G. Levine J. D. (2008). Role of interleukin-6 in chronic muscle hyperalgesic priming. Neuroscience 152, 521–525. 10.1016/j.neuroscience.2008.01.006 18280048 PMC2336107

[B10] Dumond H. Presle N. Terlain B. Mainard D. Loeuille D. Netter P.. (2003). Evidence for a key role of leptin in osteoarthritis. Arthritis Rheum. 48, 3118–3129. 10.1002/art.11303 14613274

[B11] Duruöz M. T. Turan Y. Gürgan A. Deveci H. (2012). Evaluation of metabolic syndrome in patients with chronic low back pain. Rheumatol. Int. 32, 663–667. 10.1007/s00296-010-1693-x 21132549

[B12] Fantuzzi G. (2005). Adipose tissue, adipokines, and inflammation. J. Allergy Clin. Immunol. 115, 911–919. 10.1016/j.jaci.2005.02.023 15867843

[B13] Fernández-Puente P. Mateos J. Fernández-Costa C. Oreiro N. Fernández-López C. Ruiz-Romero C.. (2011). Identification of a panel of novel serum osteoarthritis biomarkers. J. Proteome Res. 10, 5095–5101. 10.1021/pr200695p 21973172

[B14] Franceschi C. Capri M. Monti D. Giunta S. Olivieri F. Sevini F.. (2007). Inflammaging and anti-inflammaging: a systemic perspective on aging and longevity emerged from studies in humans. Mech. Ageing Dev. 128, 92–105. 10.1016/j.mad.2006.11.016 17116321

[B15] Gomez R. Lago F. Gomez-Reino J. Dieguez C. Gualillo O. (2009). Adipokines in the skeleton: influence on cartilage function and joint degenerative diseases. J. Mol. Endocrinol. 43, 11–18. 10.1677/JME-08-0131 19240195

[B16] Ha J. Y. (2011). Evaluation of metabolic syndrome in patients with chronic low back pain: using the fourth Korea national health and nutrition examination survey data. Chonnam Med. J. 47, 160–164. 10.4068/cmj.2011.47.3.160 22247916 PMC3252504

[B17] Hoy D. March L. Brooks P. Blyth F. Woolf A. Bain C.. (2014). The global burden of low back pain: estimates from the global burden of disease 2010 study. Ann. Rheum. Dis. 73, 968–974. 10.1136/annrheumdis-2013-204428 24665116

[B18] Hui W. Litherland G. J. Elias M. S. Kitson G. I. Cawston T. E. Rowan A. D.. (2012). Leptin produced by joint white adipose tissue induces cartilage degradation via upregulation and activation of matrix metalloproteinases. Ann. Rheum. Dis. 71, 455–462. 10.1136/annrheumdis-2011-200372 22072016

[B19] Johnson Z. I. Schoepflin Z. R. Choi H. Shapiro I. M. Risbud M. V. (2015). Disc in flames: roles of TNF-α and IL-1β in intervertebral disc degeneration. Eur. Cells Mater. 30, 104–117. 10.22203/eCM.v030a08 26388614 PMC4751407

[B20] Katz J. N. (2006). Lumbar disc disorders and low-back pain: socioeconomic factors and consequences. J. Bone Joint Surg. Am. 88(Suppl. 2), 21–24. 10.2106/00004623-200604002-00005 16595438

[B21] Kraychete D. C. Sakata R. K. Issy A. M. Bacellar O. Santos-Jesus R. Carvalho E. M. (2010). Serum cytokine levels in patients with chronic low back pain due to herniated disc: analytical cross-sectional study. Sao Paulo Med. J. 128, 259–262. 10.1590/S1516-31802010000500003 21181064 PMC10948061

[B22] Li W. Liu T. Wu L. Chen C. Jia Z. Bai X.. (2014). Blocking the function of inflammatory cytokines and mediators by using IL-10 and TGF-beta: a potential biological immunotherapy for intervertebral disc degeneration in a beagle model. Int. J. Mol. Sci. 15, 17270–17283. 10.3390/ijms151017270 25264742 PMC4227161

[B23] Lim G. Wang S. Zhang Y. Tian Y. Mao J. (2009). Spinal leptin contributes to the pathogenesis of neuropathic pain in rodents. J. Clin. Invest. 119, 295–304. 10.1172/JCI36785 19139561 PMC2631299

[B24] Lippi G. Dagostino C. Buonocore R. Aloe R. Bonaguri C. Fanelli G.. (2017). The serum concentrations of leptin and MCP-1 independently predict low back pain duration. Clin. Chem. Lab. Med. 55, 1368–1374. 10.1515/cclm-2016-0942 28076310

[B25] Lübbeke A. Finckh A. Puskas G. J. Suva D. Lädermann A. Bas S.. (2013). Do synovial leptin levels correlate with pain in end stage arthritis? Int. Orthop. 37, 2071–2079. 10.1007/s00264-013-1982-6 23835555 PMC3779572

[B26] Maher C. Underwood M. Buchbinder R. (2017). Non-specific low back pain. Lancet 389, 736–747. 10.1016/S0140-6736(16)30970-9 27745712

[B27] Martel-Pelletier J. Raynauld J. P. Dorais M. Abram F. Pelletier J. P. (2016). The levels of the adipokines adipsin and leptin are associated with knee osteoarthritis progression as assessed by MRI and incidence of total knee replacement in symptomatic osteoarthritis patients: a *post hoc* analysis. Rheumatology 55, 680–688. 10.1093/rheumatology/kev408 26660640

[B28] Massengale M. Lu B. Pan J. J. Katz J. N. Solomon D. H. (2012). Adipokine hormones and hand osteoarthritis: radiographic severity and pain. PLoS ONE 7:e47860. 10.1371/journal.pone.0047860 23110114 PMC3482224

[B29] Mobasheri A. Rayman M. P. Gualillo O. Sellam J. van der Kraan P. Fearon U. (2017). The role of metabolism in the pathogenesis of osteoarthritis. Nat. Rev. Rheumatol. 13, 302–311. 10.1038/nrrheum.2017.50 28381830

[B30] Pedersen L. M. Schistad E. Jacobsen L. M. Røe C. Gjerstad J. (2015). Serum levels of the pro-inflammatory interleukins 6 (IL-6) and−8 (IL-8) in patients with lumbar radicular pain due to disc herniation: a 12-month prospective study. Brain Behav. Immun. 46, 132–136. 10.1016/j.bbi.2015.01.008 25653193

[B31] Pottie P. Presle N. Terlain B. Netter P. Mainard D. Berenbaum F. (2006). Obesity and osteoarthritis: more complex than predicted! Ann. Rheum. Dis. 65, 1403–1405. 10.1136/ard.2006.061994 17038451 PMC1798356

[B32] Queiroz B. Z. Pereira D. S. Rosa N. M. Lopes R. A. Andrade A. G. Felício D. C.. (2017). Inflammatory mediators and pain in the first year after acute episode of low-back pain in elderly women: longitudinal data from back complaints in the elders-Brazil. Am. J. Phys. Med. Rehabil. 96, 535–540. 10.1097/PHM.0000000000000661 27898478

[B33] Queiroz B. Z. Pereira D. S. Rosa N. M. Lopes R. A. Felício D. C. Pereira D. G.. (2015). Functional performance and plasma cytokine levels in elderly women with and without low back pain. J. Musculosk. Rehabil. 28, 343–349. 10.3233/BMR-140526 25271196

[B34] Ranasinghe P. Mathangasinghe Y. Jayawardena R. Hills A. P. Misra A. (2017). Prevalence and trends of metabolic syndrome among adults in the asia-pacific region: a systematic review. BMC Public Health. 17:101. 10.1186/s12889-017-4041-1 28109251 PMC5251315

[B35] Robinson M. J. Edwards S. E. Iyengar S. Bymaster F. Clark M. Katon W. (2009). Depression and pain. Front. Biosci. 14, 5031–5051. 10.2741/3585 19482603

[B36] Samartzis D. Karppinen J. Cheung J. P. Y. Lotz J. (2013). Disk degeneration and low back pain: are they fat-related conditions? Glob. Spine J. 3, 133–144. 10.1055/s-0033-1350054 24436864 PMC3854598

[B37] Segar A. Urban J. Fairbank J. C. T. (2016). Adipokines and the intervertebral disc: a biochemical link exists between obesity, intervertebral disc degeneration and low back pain. Spine J. 16:S225 10.1016/j.spinee.2016.07.135 30324498

[B38] Shiri R. Solovieva S. Husgafvel-Pursiainen K. Taimela S. Saarikoski L. A. Huupponen R.. (2008). The association between obesity and the prevalence of low back pain in young adults: the Cardiovascular Risk in Young Finns Study. Am. J. Epidemiol. 167, 1110–1119. 10.1093/aje/kwn007 18334501

[B39] Suri P. Hunter D. J. Rainville J. Guermazi A. Katz J. N. (2012). Quantitative assessment of abdominal aortic calcification and associations with lumbar intervertebral disc height loss: the Framingham Study. Spine J. 12, 315–323. 10.1016/j.spinee.2012.03.033 22561175 PMC3367049

[B40] Teraguchi M. Yoshimura N. Hashizume H. Muraki S. Yamada H. Oka H.. (2016). Metabolic syndrome components are associated with intervertebral disc degeneration: the wakayama spine study. PLoS ONE 11:e0147565. 10.1371/journal.pone.0147565 26840834 PMC4739731

[B41] Urquhart D. M. Berry P. Wluka A. E. Strauss B. J. Wang Y. Proietto J.. (2011). 2011 Young investigator award winner: increased fat mass is associated with high levels of low back pain intensity and disability. Spine 36, 1320–1325. 10.1097/BRS.0b013e3181f9fb66 21270692

[B42] Vuolteenaho K. Koskinen A. Moilanen E. (2014). Leptin - a link between obesity and osteoarthritis. Appl. Prevent. Treat. Basic Clin. Pharmacol. Toxicol. 114, 103–108. 10.1111/bcpt.12160 24138453

[B43] Wang H. Cheng Y. Shao D. Chen J. Sang Y. Gui T.. (2016). Metabolic syndrome increases the risk for knee osteoarthritis: a meta-analysis. Evid. Based Complement. Alternat. Med. 2016:7242478. 10.1155/2016/7242478 27807463 PMC5078652

[B44] Wang H. Schiltenwolf M. Buchner M. (2008). The role of TNF-alpha in patients with chronic low back pain-a prospective comparative longitudinal study. Clin. J. Pain. 24, 273–278. 10.1097/AJP.0b013e31816111d3 18287835

[B45] Zhang Y. Chee A. Shi P. Adams S. L. Markova D. Z. Anderson D. G.. (2016). Intervertebral disc cells produce interleukins found in patients with back pain. Am. J. Phys. Med. Rehabil. 95, 407–415. 10.1097/PHM.0000000000000399 26495812 PMC4841745

[B46] Zhao C. Q. Liu D. Li H. Jiang L. S. Dai L. Y. (2008). Expression of leptin and its functional receptor on disc cells: contribution to cell proliferation. Spine 33, E858–E864. 10.1097/BRS.0b013e31818338e5 18978578

